# Endothelial Damage and the Microcirculation in Critical Illness

**DOI:** 10.3390/biomedicines10123150

**Published:** 2022-12-06

**Authors:** Rachael Cusack, Marc Leone, Alejandro H. Rodriguez, Ignacio Martin-Loeches

**Affiliations:** 1 Department of Intensive Care Medicine, St. James’s Hospital, James’s Street, D08 NHY1 Dublin, Ireland; 2 School of Medicine, Trinity College Dublin, College Green, D02 R590 Dublin, Ireland; 3 Department of Anaesthesiology and Intensive Care Unit, Hospital Nord, Assistance Publique Hôpitaux de Marseille, Aix Marseille University, 13015 Marseille, France; 4 Intensive Care Unit, Hospital Universitario Joan XXIII, 43005 Tarragona, Spain; 5 Institut d’Investigació Sanitària Pere Virgil, 43007 Tarragona, Spain; 6 Departament Medicina I Cirurgia, Universitat Rovira i Virgili, 43003 Tarragona, Spain; 7 Centro de Investigación en Red de Enfermedades Respiratorias (CIBERES), Instituto de Salud Carlos III, 28029 Madrid, Spain

**Keywords:** endothelium, endothelial glycocalyx, microcirculation, COVID-19, sepsis, intensive care

## Abstract

Endothelial integrity maintains microcirculatory flow and tissue oxygen delivery. The endothelial glycocalyx is involved in cell signalling, coagulation and inflammation. Our ability to treat critically ill and septic patients effectively is determined by understanding the underpinning biological mechanisms. Many mechanisms govern the development of sepsis and many large trials for new treatments have failed to show a benefit. Endothelial dysfunction is possibly one of these biological mechanisms. Glycocalyx damage is measured biochemically. Novel microscopy techniques now mean the glycocalyx can be indirectly visualised, using sidestream dark field imaging. How the clinical visualisation of microcirculation changes relate to biochemical laboratory measurements of glycocalyx damage is not clear. This article reviews the evidence for a relationship between clinically evaluable microcirculation and biological signal of glycocalyx disruption in various diseases in ICU. Microcirculation changes relate to biochemical evidence of glycocalyx damage in some disease states, but results are highly variable. Better understanding and larger studies of this relationship could improve phenotyping and personalised medicine in the future. Damage to the glycocalyx could underpin many critical illness pathologies and having real-time information on the glycocalyx and microcirculation in the future could improve patient stratification, diagnosis and treatment.

## 1. Introduction

Publication of research into the endothelial glycocalyx and microcirculation has increased exponentially in the last decade. Our understanding of the glycocalyx has changed from that of it being a ubiquitous, jelly-like layer to taking an active role in the interaction between the intravascular and interstitial space. The glycocalyx plays a role in chronic inflammation, diabetes, trauma, sepsis and ischaemia-reperfusion injury. The microcirculation is deranged in approximately 17–20% of a heterogenous population of patients in ICU [1,2]. Microcirculatory derangements can persist despite adequate macrocirculation corrected by vasopressors or transfusion [3]. In those that the microcirculation cannot be recruited or restored, morbidity and mortality is increased.

The glycocalyx is a carbohydrate rich layer responsible for maintaining not only the oncotic pressure and barrier function within the circulatory system, but also antithrombotic and inflammatory signalling functions. It covers the luminal surface of endothelial cells throughout the vascular network, but changes between tissues depending on its primary function [4].

Made up of proteoglycans and glycosaminoglycans, the glycocalyx creates a strong negative charge within the blood vessels to repel large molecules from escaping into the tissues. In vitro experiments have shown that the damaged glycocalyx becomes sensitised to atherogenic and inflammatory mediators. This sensitisation causes release of chemotactic molecules and by increasing the production of inflammatory mediators, it precipitates a cytokine storm. Circulating components of the inflammasome bind to glycocalyx receptors in a cycle of increasing inflammation. This leads to widening of gap junctions and relaxing of the barrier between the lumen and the interstitium, marked by shedding of glycocalyx molecules. The functionality of the endothelium is dependent on remaining intact with all relevant sidechains and molecular structures in place. Glycocalyx shedding and deterioration leads to loss of endothelial integrity and function [5,6]. 

Multiorgan dysfunction syndrome including encephalopathy, acute kidney injury, acute liver injury, coagulopathy and acute respiratory distress syndrome can all be associated with endothelial damage. Mediators released in sepsis act on the glycocalyx to produce a global response. By assessing the microcirculation in patients with sepsis, we can better understand the changes that occur in response to insults such as endotoxin or oxidative stress and link these back to markers of endothelial damage. The microcirculation has been described as the largest organ in the body, comprising the capillaries and venules <20 µm where red blood cells (RBCs) often travel in single file. It is where the transfer of vital oxygen from the circulation to the tissues takes place and so it is where our resuscitative efforts should be focused. Some previous studies have demonstrated the usefulness of the microcirculation as a prognostic tool in ICU patients [7]. Sidestream dark field (SDF) imaging has been assessed as a potential prognostic tool to guide therapy. New technologies are emerging that would allow clinicians to directly examine the EG, a potential huge step forward in personalised medicine and point-of care diagnostics.

This narrative review explores the relationship between endothelial damage, how the glycocalyx relates to the clinically observable microcirculation and how we can use this connection to improve patient outcomes. Personalised medicine revolves around our ability to treat each patient and their unique pathology or phenotype individually. 

### Endothelial Glycocalyx in Clinical Practice

The loss of glycocalyx function, defence and configuration impairs vessel mechano-transduction, platelet and leucocyte adhesion to the endothelial surface and causes invasion of the vascular compartment with fluid and plasma proteins [8]. At the level of myocardium, it was found that endothelial leak was responsible for swelling in the subendothelial space, resulting in the compression of the capillary lumen and leading to oedema and myocardial dysfunction [9]. Continuation of inflammation increases availability of leucocytes to adhesion molecules by attacking the surrounding EG. Inflammatory mediators directly influence the glycocalyx and its constituents and adjust the structure. Degranulation of activated inflammatory mediators such as mast cells and macrophages release reactive oxygen species (ROS), reactive nitrogen species (RNS) that also participate to the degradation of the EG [10]. Neutrophils are the most abundant circulating cells in the human body and release proteases that damage the glycocalyx also.

Glycocalyx dysfunction can occur in any organ and so can be recognised in several clinical conditions. One of the first syndromes that had recognised glycocalyx damage was diabetes. The first studies that quantified the glycocalyx found that patients with diabetes type 1 had a reduced volume of glycocalyx by 500 mL, compared to healthy subjects [11]. Glycocalyx involvement has been found in cardiovascular disease, including hypertension, stroke and left ventricular remodelling after myocardial injury, as well as cancer, renal failure, diabetes, obesity, cognitive impairment, pre-eclampsia, advanced age and COVID-19 [12,13,14,15,16,17,18,19,20,21,22]. An analysis of the ProCESS trial patients showed that elevated markers of glycocalyx damage in blood, angiopoietin-2 (Ang-2), vascular endothelial growth factor-1 and -2 (VEGF) and soluble fms-like tyrosine kinase (sFLT-1) were associated with increased 60-day in hospital mortality at baseline and at timepoints 6 and 24 h [23]. 

The widespread pathological effects of SARS-CoV-2 infection across various organ systems made a strong case for a glycocalyx driven disease. Before widespread effective vaccination campaigns many infected patients required hospitalisation and up to 43% who required invasive mechanical ventilation, after failure of non-invasive ventilation, and ICU support would die [24,25]. Involvement of the angiotensin converting enzyme-2 (ACE-2) receptor, the prevalence of systemic microthrombi, and large vessel thromboembolic phenomena suggested a vascular pathology. The presence of this receptor throughout not only the pulmonary epithelia but the renal, vascular endothelium and arterial smooth muscle cells can explain these features [26]. Patients with COVID-19 had higher serum concentrations of the glycocalyx marker syndecan-1 but had improved microcirculation at Day-2 of admission than non-COVID sepsis patients in one observational study of 28 ICU patients [27]. The authors concluded that the worse glycocalyx damage with conserved microcirculation could represent a new sub-phenotype of septic shock with endothelial remodelling. There was also evidence of persistent endothelial damage months after infection that was attributed to oxidative stress, endothelial and vascular dysfunction [22]. The MYSTIC study demonstrated not only higher circulating plasma markers of endothelial damage but also reduced small capillary density and an association of increased perfused boundary region (PBR), glycocalyx damage and outcome [28]. The authors showed that investigational biomarkers of glycocalyx damage ADAMST-13 and VEGF were better correlated with outcomes than CRP and IL-6. Although this study included only a small number of patients and should be considered as hypothesis generating, the results are compelling. A large trial comparing moderate, severe COVID-19 and sepsis and septic shock ICU patients would be intriguing, though possibly no longer feasible post-vaccine. 

The multisystem inflammatory syndrome in children (MIS-C) frequently associated with shock emphasises the multisystem nature of the disease [29]. Endothelial involvement leads to disease sequelae in almost every organ [22]. The cardinal features of MIS-C are hyperinflammation and cytokine storm, features also recognised in the adult illness [30]. Distributive shock results from endothelitis and systemic capillary leak while there is also potential cardiogenic shock through myocardial oedema [22,31]. In a study including COVID-19 paediatric patients [29], the authors found a significant negative correlation between left ventricular ejection fraction (LVEF) and Ang-2 (*p* = 0.01). Varga et al. also found extensive endothelial cell involvement with macrophage activation, capillary leak and micro-thrombosis [32].

## 2. Measuring the Glycocalyx

Despite our increasing knowledge about the EG, it remains remarkably difficult to assess. The glycocalyx is composed of sugar and proteins that are reactive with many common laboratory fixation methods [33]. It was first visualised by staining with ruthenium red, a substance with high affinity for the acidic mucopolysaccharides, generating detectable electron density visible with an electron microscope [33,34]. Ruthenium red however is a relatively large molecule and there were concerns that its charge induces conformational change in the EG, leading to inaccurate characterisation of the glycocalyx structure [33]. Efforts were made with smaller molecular dyes (alcian blue) but other techniques were developed as classic perfusion fixation was possibly removing side chains and structural components of the system being examined.

The components of the glycocalyx are constantly being generated and shed, so damage to the glycocalyx can be assessed by measuring the concentrations of circulating endothelial components in plasma. The most reliable and widely used is syndecan-1, however levels of heparan sulfate, chondroitin sulfate, endocan and hyaluronan have also been used. Syndecan-1 is a member of the family core glycocalyx proteoglycans varying from 25–40 kDa that is measured by ELISA. They have a single-span transmembrane domain connecting to the cell membrane. Syndecans have 4 subtypes that each binds a different sidechains, either 3–5 chains of heparan sulfate or chondroitin sulfate [35]. As the syndecan sidechains are shed from the endothelium they can be measured in circulation.

Soluble shed portions of syndecans can be used as biomarkers as the process of shedding is specifically regulated under disease conditions [36]. Leukocyte-derived proteases and growth factors, associated with cellular injury or wound healing, can initiate shedding [36]. Thus, shed syndecans are found in inflammatory fluid and associated with tissue damage in a variety of disease and critical illness. During inflammation the total expression of syndecans is increased [37,38]. SDC1 plays an important role in leukocyte adhesion, vascular permeability and mechanosensation [39]. It has been studied as a biomarker in a wide range of diseases including kidney disease, heart failure and as an indicator after major surgery [40]. Soluble SDC1 is found in the peripheral blood of patients with sepsis, ischemia-reperfusion injury and graft-versus-host disease [41,42,43,44]. 

SDC2 plays a role in endothelial damage and vascular dysfunction when endothelial cells are damaged [45]. Inflammatory signals such as hypoxia and TNF-α increase expression of SDC2 in fibroblasts, endothelial cells and intestinal epithelia [46,47]. SDC3 is the largest of the syndecans but is the least studied and understood. It has been implicated in alzheimer’s disease, human immunodeficiency virus-1 (HIV) disease, angiogenesis and arthritis [48]. Cleaved portions of SDC3 disassemble endothelial cell junctions in the lung which has implications for sepsis and diseases where thrombin is activated [49]. Knockout experiments show that lack of SDC1 or SDC4 increases the inflammatory response, possibly indicating an anti-inflammatory role as well [50,51]. SDC4 is involved the development of fibrosis in the lung during inflammation [52,53]. Levels of SDC4 increase in acute pneumonia and correlate with pneumonia severity, indicating it could be a useful biomarker in these patients [54]. 

**Figure 1:**
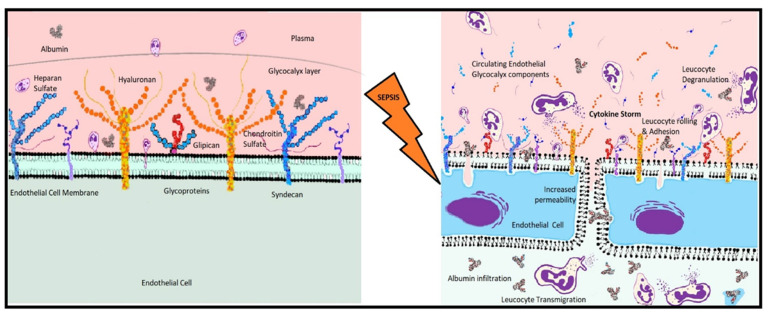
Endothelial cell layer under normal and septicconditions; Activation of leucocytes by binding of, e.g., LPS, causes degranulation, release of reactive oxygen species, reactive nitrogen species and cytokine storm which activates chemotactic actors as well.

Glycosaminoglycans are disaccharide polymers of L-iduronic acid, D-glucuronic acid or D-galactose linked to either D-N-acetyl galactosamine or D-N-acetylglucosamine [35]. Proteoglycans, mainly heparan sulfate, provide abundant binding sites for circulating mediators courtesy of their various sulfation combinations [55]. Heparan sulfate also performs vital antioxidant function binding superoxide dismutase to protect the glycocalyx from oxidative stress. This mechanism is challenged in sepsis and septic shock, leading to glycocalyx damage and extravasation of plasma proteins and fluid into the subendothelial layer (Figure 1). Reduced concentrations of heparan sulfate in serum subsequent to exposure to damaging enzymes increase coagulation and micro-thrombosis, increase adhesion molecule expression and increase leucocyte tracking along the glycocalyx [35]. In an observational study of 38 patients, blocking heparanase, an enzyme that targets heparan sulfate, with heparin eliminated glycocalyx damage in vitro [56]. Hyaluronan is attached to the cell surface via CD-44 receptor; it is not a core protein but contributes to the glycocalyx volume by its length and by binding water ~10,000 times its mass [5].

A prominent drawback of using plasma measurements is their dependence on renal clearance, which can be altered in critical illness, impacting on reliability of these tests [57,58]. Another drawback is that chronic inflammatory state also leads to increase in circulating endothelial components [6,10,35,59,60]. Metabolic, vascular and surgical diseases such as diabetes, atherosclerosis, hypertension, ischaemia reperfusion injury and trauma result in increased numbers of plasma glycosaminoglycans that correlate to inflammatory marker serum concentrations. 

Other biomarkers for endothelial damage include hyaluronic acid, angiopoietin-2, VEGF and vonWillebrand Factor cleaving protease ADAMTS-13, soluble thrombomodulin and soluble angiopoietin receptor (TIE-2) [28,61,62]. Ang-1 and Ang-2 are in opposition to one another, their action on the glycocalyx being mediated by the TIE-2 receptor. Ang-2 is the leakage inducing form and is raised in systemic inflammatory syndromes, indicating glycocalyx damage [63]. In vitro studies on human sepsis sera showed that the TIE-2 pathway regulates the glycocalyx in sepsis in a non-redundant fashion. When endothelial cells were incubated with sepsis serum and TIE-2 pathway inhibitors, the damage to glycocalyx was prevented [63,64]. 

## 3. Visualising the Microcirculation and the Endothelial Glycocalyx

Following observations that 40 kDa dextrans equilibrate with the EG, efforts to visualise and quantify the glycocalyx began by comparing dilution of fluorescently labelled RBCs to dilution of 40 kDa dextran at the time of injection [65]. Studies of the glycocalyx in cremaster muscle of mice found that the glycocalyx repelled RBCs and slowed plasma while being compressed by passing leucocytes it serves as both a barrier and a gateway to the tissues [65,66]. Visualising the glycocalyx in vivo and how it behaves in clinical practice has become more important as we come to understand its importance. The development of intra-vital microscopic techniques has transformed this area of practice. 

Developed to examine the movement of RBCs within the circulation, Orthogonal Polarisation Spectroscopy (OPS) allowed clinicians to have a view of the microcirculation in clinical practice. The most recent iterations of this technology—SDF and Incident Dark Field (IDF) imaging, have improved the clinical applicability of the microcirculation. The implications of damage to the microcirculation in a variety of diseases in ICU has been studied since these devices have been available [67,68,69].

As techniques have improved, our field of view has grown. The most recent descendant of the OPS devices, the IDF microscope has an increased field of view and improved contrast to better identify cells and perfused capillaries [70]. In the past, the image would be manually divided into sections and the boundaries of vessels individually marked out and perfused vessels counted individually. This is presently done objectively by a piece of software, AVA (Microvision Medical, Amsterdam, The Netherlands). Similarly, because the device uses 540 nm light in a dark field created by circumferential light-emitting diodes (LEDs), it highlights the RBCs themselves [71]. While this gives excellent information about availability of haemoglobin and functional capillary perfusion, the glycocalyx that controls the flow remains invisible. 

The PBR is the area at the limit of a blood vessel where RBCs can permeate, representing the luminal aspect of the glycocalyx accessed by the RBCs in circulation. It is quantified by observing the microscopic lateral motion of the cells under SDF microscopy in combination with proprietary Glycocheck™ 5.2 software (Capiscope handheld, KK Research technology Ltd., Honiton, UK). If the glycocalyx is shed or disturbed, the lateral motion of RBCs increases so PBR has an inverse relationship to glycocalyx thickness. 

The GlycoCheck™ system makes it possible to calculate the degree of lateral motion of RBCs within small capillaries [72]. The reliability of this system has been established both due to its interobserver consistency and accessibility to all clinical staff as a potential standard monitoring tool [14,73]. The success of the GlycoNurse study established the system’s potential to bring the microcirculation from the research realm into daily clinical practice in a busy Emergency department environment [74]. The GlycoCheck system is a great leap forward from other in vivo glycocalyx measurement methods such as atomic force microscopy and microparticle image velocimetry (μ-PIV), used on animal models in laboratory conditions. The PBR may be elevated in microvascular thrombosis, inflammation or sepsis, and it has been used to visualise the glycocalyx in vivo [75]. 

The NOSTRADAMUS study used RBC velocity measurements together with PBR thickness to improve discrimination between patients with sepsis and healthy controls [76]. This study showed that the PBR tends to increase when the velocity of RBCs decreases, indicating increased permeability and porosity of the glycocalyx in an environment of reduced shear stress. 

### 3.1. The Sublingual Target Region

The sublingual region is most commonly used area to study the microcirculation because of the proximity to the lingual artery as a branch of the external carotid artery, giving the clinician insight into the reactivity of the central circulation. However, other vascular beds such as the intestinal bed, renal bed, conjunctival and peripheral muscular microvasculature have also been used to study the microcirculation. 

The sublingual region is the most clinically accessible however, its reliability relies on how representative it is of all vascular beds. In a pig model, where septic cholangitis was induced by *Escherichia coli* into the common bile duct, OPS imaging of the intestine and the sublingual region correlated well in timing and specific observable microcirculation changes [77]. A prospective study of patients with sepsis after formation of an intestinal stoma correlated OPS images from within the stoma with sublingual images [78]. This study found no relationship between the two regions on postoperative day 1, but the relationship normalised by day 3. MFI in the stoma of the sepsis group was significantly lower than healthy controls and the non-septic new stoma group. Sublingual region MFI at day 1 correlated well with macrohaemodynamic measures such as sequential organ failure assessment (SOFA) and length of stay, this relationship was not significantly related on day 3. 

However, another clinical observational study of postoperative ostomy patients before and after fluid challenge found dissociation of the intestinal and sublingual microvascular beds [79]. In response to a fluid challenge on the first postoperative day, the sublingual but not the intestinal microcirculation showed increased RBC velocity. This study did not perform follow up imaging to see if this dissociation resolved or persisted. A study of patients undergoing gastrointestinal surgery were assessed by SDF imaging of their bowel and sublingual region intraoperatively. Studying the sublingual region allowed for more stable image acquisition, less pixel loss and faster image acquisition [80]. There was good correlation of MFI, PVD and TVD between sublingual and gastrointestinal microcirculation. 

### 3.2. Near Infrared Spectroscopy and the Microcirculation

Near infrared spectroscopy (NIRS) is a non-invasive tool that measures microvascular reactivity by oxygenation in muscle, commonly the deltoid or thenar eminence. Studies on patients with sepsis have associated low thenar eminence saturations with poor outcome in sepsis. Using a vascular occlusion test in the forearm, microcirculation reactivity can be assessed by analysis of tissue saturations changes during an ischaemic challenge. This illustrates oxygen extraction by tissues and reactivity of the microvascular bed. A meta-analysis of static and dynamic NIRS and mortality in sepsis found that septic patients had lower tissue saturations, decreased reperfusion slope and lower reperfusion hyperaemic maximum tissue saturation. These results were also associated with higher mortality in septic patients [81]. A prospective study of patients in septic shock found an association between septic shock and lower initial tissue saturations, impaired occlusion slope and recovery slope, implying microcirculation dysfunction. SOFA score was also associated with recovery slope in the septic shock cohort with AUC 0.81, meaning NIRS could be an interesting non-invasive prognostic monitor in the future [82]. Moreover, other studies ICU found microcirculation failure measured by NIRS predicted mortality [83]. 

## 4. Clinical Applications of Microcirculation Monitoring

**Figure 2:**
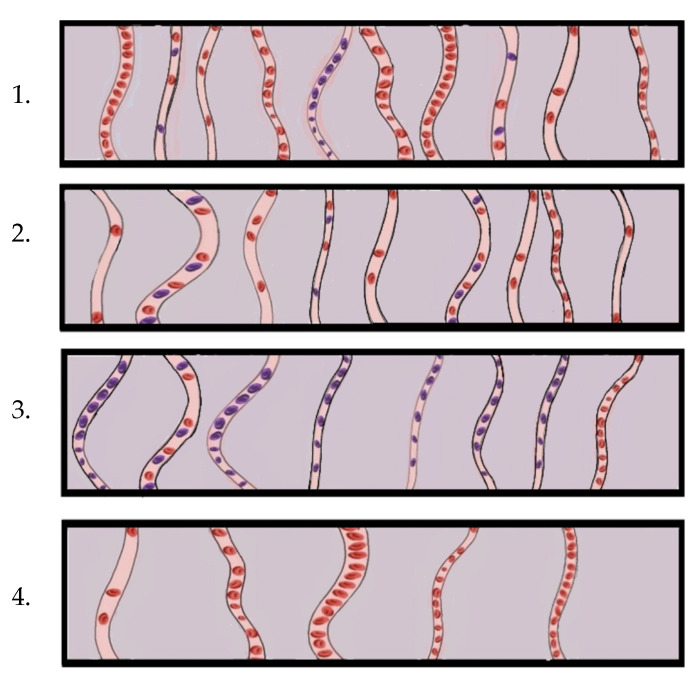
Observable microcirculation changes in the sublingual region. 1. Heterogenous flow; microthrombi in vessels next to regions of hyperdynamic flow, resulting in heterogeneous oxygen delivery; 2. Haemodilution; adequate microvascular density but low haematocrit results in low oxygen delivery; 3. Tamponade; increased venous back pressure leading to stagnation and sluggish flow; 4. Oedema; increased distance between vessels increases oxygen diffusion distance [87].

Systemic inflammation causes specific changes in the appearance of the sublingual microcirculation. Cellular hypoxia resulting from loss of coherence between the macro and the microcirculation is due to specific changes in the distribution of flow in the microcirculatory vessels [84]. Cellular hypoxia is a result of microcirculation failure and not the cells altering the flow distribution in response to hypoxia [85,86]. These changes have been characterised as being of four types; heterogeneous, haemodilution, constriction/tamponade and oedema formation [87] (Figure 2). Septic patients’ capillaries can be blocked with microthrombi, next to perfused vessels. This leads to differential perfusion of cells in tissue and increased hypoxic metabolism. Haemodilution increases the distance between oxygen rich RBCs in the capillary and the respiring cells in the tissue, this increased diffusion distance is similar to the mechanism of cellular hypoxia following interstitial oedema formation. Finally, systemic variables such as increased vascular resistance or increased venous back pressure can cause tamponade and flow restriction, leading to sluggish flow or complete stagnation [84].

### 4.1. Haemorrhage and Trauma

Patients with traumatic haemorrhage experience microcirculation effects, including reduced MFI and PPV, that is greater in patients with higher sequential organ failure assessment (SOFA) score and lasts for at least 72 h [88]. A prospective observational study of patients with traumatic haemorrhagic shock found that those with multiple organ dysfunction (MOD) had lower MFI and PVD at day 0 but similar cardiac index to patients without MOD [89]. This study showed that the phenomenon of haemodynamic incoherence is preserved in different forms of shock and supported microcirculation monitoring in trauma as a treatment target and prognostic indicator. As part of this study, safety and feasibility of performing sublingual microscopy in trauma patients was also assessed. The authors concluded that emergency department monitoring of sublingual microcirculation is safe and appropriate given the valuable prognostic information available [90]. SDF monitoring did not interrupt clinical management and it was possible to acquire high quality images. Another observational study of 17 trauma patients monitored syndecan-1 and thrombomodulin to assess endothelial damage and correlate it to microcirculation dysfunction. They found that, compared to healthy controls, patients had increased syndecan-1 associated with worse MFI, TVD, PVD, PPV and heterogeneity index as well as higher thrombomodulin associated with worse PPV and MFI [91]. The association between endothelial damage and microcirculation failure was conserved over the 50 h post-injury study period.

### 4.2. Cardiac Component 

The microcirculation is also altered in patients with cardiogenic shock. Patients with cardiac failure or cardiogenic shock have lower perfused small vessel density and lower PPV than controls [92]. In a pig model of ventricular fibrillation and precordial compression, the animals in whom the microcirculation improved with compressions had a greater proportion of return of circulation [93]. This is conserved in humans also. A sub-study of the culprit lesion-only percutaneous coronary intervention versus multivessel percutaneous coronary intervention in cardiogenic shock (CULPRIT-SHOCK) trial found that normotensive patients with microcirculation failure were at increased risk of 30-day all-cause mortality than normotensive patients with preserved proportion perfused capillaries and perfused capillary density [94]. A randomised study of intra-aortic balloon pump therapy for cardiogenic shock complicating acute myocardial infarction published a sub-study of glycocalyx markers over the first 48 h post-percutaneous coronary intervention (PCI). This sub-study of 184 patients found that survivors at day 30 had lower levels of syndecan-1 and a trend towards lower levels of heparan sulphate [95]. Univariate logistic regression and multivariable adjustment found syndecan-1 to be an independent predictor of mortality. Thirty-three patients with cardiogenic shock on veno-arterial extracorporeal membrane oxygenation (VA-ECMO) were recruited to an observational study of the microcirculation over the course of ECMO. The 19 patients that survived demonstrated higher small vessel density, perfused small vessel density and MFI, than non-survivors [96]. This study also identified a novel biomarker that correlated with microcirculation variables as well as 30-day hospital mortality in a multivariate logistic regression model. Another study of ECMO and microcirculation found that the sublingual microcirculation could be used as a useful predictor of those patients that will successfully wean from ECMO within 48 h. Those patients who maintained their TVD and PVD during a reduction of ECMO flow by 50% were more likely to wean successfully from ECMO support [97]. The results of these studies demonstrate the importance of clinical monitoring of sublingual microcirculation in various types of shock in ICU.

One area that has shown an association between the glycocalyx and the microcirculation is patients undergoing cardiopulmonary bypass (CPB) [98,99]. Studies in this field have shown associations between glycocalyx marker serum concentrations and visualised microvascular changes during CPB. The authors postulate that intraoperative glycocalyx damage with CPB plays a role in microcirculation perfusion dysfunction in the following postoperative days [99]. This is supported by other studies associating CPB and microcirculation changes with plasma markers of glycocalyx damage. Measuring glycocalyx degradation marker serum concentrations, together with sublingual PBR, during CPB with heparin coated or phosphorylcholine coated bypass circuits found a correlation between phosphorylcholine circuits and microvascular changes intraoperatively. This study showed the close association between the glycocalyx damage and the microcirculation [98]. Another study showed that endothelial damage markers persist in blood up to 72 h following CPB [100]. It is possible that there is a spatiotemporal disconnect when attempting to replicate similar studies in ICU and patients with sepsis. The timing of insult and appearance of measurable markers of glycocalyx damage are not so discreet as a single insult such as CPB. This study used syndecan-1, heparan sulfate and hyaluronan to measure endothelial response to CPB. Syndecan-1 has been shown to correlate closely with in vivo PBR and was also in close agreement with glycocalyx thickness measured by atomic force microscopy (AFM), a technique that closely measures nano-mechanics of the glycocalyx [24,53].

### 4.3. Sepsis and Septic Shock

The glycocalyx is degraded in sepsis, as circulating inflammatory mediators cleave hyaluronic acid and heparan sulfate through oxidation reactions with ROS [10]. Cell culture experiments have shown that following enzymatic degradation, full recovery of the glycocalyx occurs over 72 h [101,102]. These experiments were carried out in vitro and not under septic conditions. Thus, in reality, recovery could potentially take even longer for the glycocalyx after an initial but persisting insult. This time delay could explain the difference between macro- and microcirculation recovery time. One animal model study found that following a single bolus of enzymatic degradation with hyalurodinase, heparinase or tissue necrosis factor-α (TNF-α) it took 7 days for mouse cremaster muscles to regenerate meaningful endothelial architecture [103]. 

Raised Ang-2, a marker of increased glycocalyx permeability, correlates with microvascular injury demonstrated by sublingual PBR measurements in ICU patients [56]. The MYSTIC study showed higher sublingual PBR in the 40 septic patients compared to controls as well as higher Ang-2 serum concentrations [28]. Developing their cell culture model, they showed that the glycocalyx damage in vitro from sepsis patients sera correlated well with sublingual PBR values. A prospective cohort study of 66 patients found a statistically significant increase in median Ang-2 levels between patients with sepsis vs. those with septic shock (19 ng/mL vs. 11 ng/mL, *p* = 0.01) [104]. Ang-2 correlated with illness severity scores, IL-6, lactate and significantly correlated with in hospital mortality. A prospective study of 28 patients found that increased PBR was correlated inversely with TVD and PVD, demonstrating a link between glycocalyx stiffness and microcirculation impairment in sepsis [105]. 

Impairment of the sublingual microcirculation of sepsis and septic shock patients has been linked to MODS, severity of sepsis and mortality [3,67]. De Backer et al. reported that the microcirculation is better in the later phases of sepsis than earlier [3]. Importantly, if mean arterial pressure (MAP) is below 60 mmHg then the microcirculation is disrupted in almost all patients [106,107]. However, another report showed that if the microcirculation is measured early in the course of sepsis it is more likely to respond to treatment than those who have impaired microcirculation 48 h after admission [98]. Even once the microcirculation has been corrected, it remains disrupted in about 50% of patients who are then at an increased risk of death. Those patients whose microcirculation responds to treatment and recovers within 48 h have associated increased survival [108]. Dubin et al. showed that when the MAP is augmented with noradrenaline, there were no changes in sublingual microcirculation (MFI, PPV) for MAP value of 65, 75 or 85 mmHg. In fact, they showed a trend towards an inverse relationship between the sublingual perfused capillary density and MAP [109]. The MAP target of 65 mmHg in septic shock has previously been found to be sufficient, although its relationship to the adequacy of the microcirculation deserves more attention in the future [110].

## 5. Haemodynamic Coherence and Personalised Treatment in ICU

Restoration of macro-haemodynamic stability does not reliably re-establish the microcirculation [111]. This has been dubbed haemodynamic coherence and ICU research and resuscitation should aim to understand and improve it [87,112]. A physiological state where despite the gross improvement of macrohaemodynamic markers such as blood pressure and heart rate, the microcirculation remains impaired [87]. ICU resuscitation relies on appropriate restoration of cellular respiration. Haemodynamic coherence represents the potential downfall of many large trials of heterogeneous groups of ICU patients [113].

Reclassification of acute respiratory distress syndrome (ARDS) biological and clinical phenotypes has increased prognostic and predictive enrichment by defining homogenous groups within this particular disease [114]. By recognising separate cohorts within large heterogeneous groups, treatments can be targeted at those that will benefit most. Those at increased risk of a particular adverse outcome may be more likely to benefit from a certain intervention, increasing a study’s power or a biologically homogenous group may be more likely to benefit from an intervention targeting a specific biological mechanism. For example, the PaO2:FiO2 ratio <150 mmHg cut-off was used in ACURASYS and PROSEVA to show benefit of muscle blockade and proning in the most severe cases of ARDS [115,116]. Similarly, by recognising fluid responders and non-responders, treatments for sepsis can be studied more effectively.

Recognising the changes in the microcirculation in different pathological states could help to identify homogenous patient cohorts [67,117,118]. Studies of the microcirculation response to RBC transfusion have shown a heterogeneous response of groups of patients clinically diagnosed as sepsis or septic shock [119]. These results indicate the existence of subsets of microcirculation changes that may respond differently to therapies. Previous studies have shown that despite individual haemodynamic incoherence, sepsis induced dysfunction of the microcirculation can recover following resuscitation of arterial pressure.

## 6. Prognostic Value of Glycocalyx Damage in Critical Illness

The connection between glycocalyx degradation, microvascular parameters and systemic clinical markers has been difficult to identify. In non-septic ICU patients only a weak correlation could be found between syndecan-1 and the glycocalyx thickness measured in the sublingual region [120]. Rovas et al. found that PBR, MFI and PPV correlated with measures of critical illness including mean arterial pressure, CRP, IL-6 and procalcitonin (PCT) [121]. They also found an association with systemic inflammatory response (SIRS) and SOFA score. The interest of this study was to attempt to draw together disparate prognostic indicators and to associate bedside microcirculation assessment with glycocalyx function. However, another study showed that PBR and syndecan-1 serum concentrations did not correlate with microcirculation variables. The NOSTRADAMUS study attempted to link the macro and microcirculation by suggesting the Microvascular Health Score (MVHS). The MVHS depends on the correlation Rovas et al. found between flow dependent capillary density and SOFA [76]. This study used RBC velocity measurements together with PBR thickness to improve discrimination between patients with sepsis and healthy controls [76]. One of the largest biomarker trials conducted was the Protocolized Care for Early Septic Shock (ProCESS) randomised controlled multicentre trial [122]. An analysis of 1341 of these patients showed that elevated markers of endothelial permeability in blood, angiopoietin-2 (Ang-2), vascular endothelial growth factor-1 and -2 (VEGF) and soluble fms-like tyrosine kinase (sFLT-1) were associated with increased 60-day in hospital mortality at baseline and at timepoints 6 and 24 h [23]. Though no difference was found between the treatment groups of the trial, there was a significant difference in mortality according to baseline serum concentrations of endothelial markers. A systematic review of 17 studies investigating the relationship between markers of glycocalyx degradation and outcomes in sepsis showed that concentrations of syndecan-1 and endocan were higher in patients who died, developed MODS or experienced renal failure [123]. In a prospective study of 21 sepsis patients PBR correlated positively with plasma concentrations of Ang-2 (R = 0.52, *p* = 0.03) but not with APACHE, SOFA, lactate or syndecan-1 [124]. Increased endothelial permeability can be clinically detected as microalbuminuria-urinary creatinine ratio (MACR), as a result of glomerular inflammatory injury. MACR is an early marker of sepsis and a marker of severity that correlates with Acute Physiological Score II (APACHE), SOFA, Simplified Acute Physiology Score II (SAPS) [125,126,127].

## 7. Restoration of Glycocalyx Function

### 7.1. Fluid Therapy and the Glycocalyx

Protocolised treatment for sepsis has focused on fluid resuscitation to restore the circulating volume [128,129,130]. Distributive shock in sepsis, secondary to glycocalyx damage, leads to reduced systemic vascular resistance and hypotension. Guidelines recommend treatment with fluid bolus, aiming to optimise the cardiac preload. Aggressive fluid resuscitation and the use of hyper-oncotic solutions may actually damage the glycocalyx further in disease states [131,132]. The results of large trials of fluid resuscitation techniques and their failure to demonstrate benefit or in some cases cause harm may stem from our fundamental misunderstanding of the function of the endothelium [133]. Large studies such as The FINNAKI trial with over 600 ICU patients found that vascular adhesion protein 1 (VAP-1) decreased and IL-6 increased with increasing amounts of administered fluid [132]. This study also found that 90-day non-survivors had higher levels of circulating Syndecan-1 and soluble Thrombomodulin (sTM) compared to those who survived. The log Syndecan-1, log sTM and logAng-2 were significantly associated with an increased risk for 90-day mortality [132]. Moreover, other studies conducted in patients with sepsis patients found that for each 1 L of intravenous fluids administered there was a significant rise in heparan sulfate, independent of age and clinical severity, suggesting increased glycocalyx destruction [131].

Recent studies demonstrating that “restrictive” or “conservative” fluid resuscitation strategies are safe and non-inferior to traditional protocols have challenged the idea that more fluid is better in sepsis [134,135,136]. Excessive fluid resuscitation can induce and increase endothelial glycocalyx degradation [6,137,138]. The association of excess fluid and poor outcomes has been examined in several studies [139,140,141,142]. A study comparing prolonged infusion to fluid bolus found no difference in plasma markers of endothelial damage in ICU patients [143]. Atrial natriuretic peptide (ANP) causes degradation of the glycocalyx and is released in response to volume loading with fluid in healthy patients preoperatively. Raised ANP concentrations were associated with increased serum concentrations of hyaluronan and syndecan-1 showing glycocalyx shedding in one observational clinical study [144]. These markers of glycocalyx damage were found only in those who had been volume loaded but not who had received normovolemic fluid replacement, which should encourage further study and suggests a damaging effect of fluid resuscitation. More work to correlate the glycocalyx status of patients with bedside diagnostics and parameters is warranted.

One study, using both a caecal ligation and puncture (CLP) and LPS-induced pulmonary inflammation mouse model showed that the pulmonary glycocalyx deteriorates 8 h following sepsis induction. Interestingly this study also found a protective effect of 6% hydroxyethylstarch (HES) that reduced plasma concentrations of glycocalyx damage and conserved glycocalyx thickness to reduce vascular permeability [145]. HES has had its European licence revoked, because of studies demonstrating a possible link between HES and acute renal injury [146]. The Starling model and its explanation of colloid osmotic forces leads us to the conclusion that supplemental albumin should improve intravascular volume and recruit interstitial fluid. The most recent proposed mechanism suggests that the sub-glycocalyx, being protected by the negative charge of the glycocalyx, prevents transcapillary flow, rather than the luminal colloid osmotic pressure. However, this does not appear to be strictly true colloids do tend to improve hypovolemia in spite of the revised Starling model [147]. In animal studies, fluid resuscitation with albumin reduced glycocalyx permeability and leucocyte adhesion, similar to fresh frozen plasma (FFP), also lowering syndecan-1 levels [148]. Glycocalyx effects have been recognised in a recent review to be maximised by resuscitation with plasma and albumin are superior to crystalloid and colloid [149]. A randomized, multi-centre study of abdominal surgery patients receiving crystalloid, 20% albumin or 20% albumin and dexamethasone intra-operatively found no difference in syndecan-1 levels post-operatively [150]. Albumin acts on the glycocalyx primarily through sphingosine-1-phosphate (S1P) and the potential role of this molecule in resuscitation on the glycocalyx has also been explored recently [151]. In vitro shock models exposed to S1P, albumin + S1P or carrier protein + S1P found endothelial damage repaired best by the carrier protein + S1P, which raises the possibility of new treatments for endotheliopathy for future research [152].

### 7.2. Corticosteroids and the Glycocalyx

Dexamethasone became important to treat COVID-19 in ICU patients during the pandemic [153]. The effect of dexamethasone on the endothelium has been explored in other studies, with effects possibly mediated through inhibition of nitric oxide synthase [154]. This animal model of acute lung injury secondary to endotoxin injection showed a possible mechanistic link between dexamethasone and one of the main contributors to glycocalyx damage. Experimental studies support the possible endothelial protective effects of hydrocortisone, reducing vascular leak following injury due to systemic inflammation or injury [155,156,157]. A guinea-pig heart model of ischaemia-reperfusion injury found that the benefit of hydrocortisone relies directly on its effect on the glycocalyx, as this mechanism was both visualised under electron microscopy and measured by syndecan-1, heparan sulfate and hyaluronan shedding, rather than systemic anti-inflammatory effects [156].

The effect of dexamethasone in repairing cerebral endothelium after haematoma and protecting the vascular endothelium from statin induced damaged also provides evidence for this anti-inflammatory glucocorticoid acting on the glycocalyx [158,159]. In an observational study of patients with COVID -19, one group found that those 63% of patients that received dexamethasone had improvements in oxygenation and exhibited lower levels of Ang-2, intercellular adhesion molecule-1 (ICAM-1) and soluble Tie-2 receptor. This study suggests that disease severity is related to endothelial damage and also that this can be modified with dexamethasone administration [160]. Sepsis guidelines highlighted a number of studies, including systematic reviews on randomised controlled trials underlining conflicting evidence regarding the use of hydrocortisone in septic shock [128]. However, they did emphasise the potential benefit of using hydrocortisone in septic shock patients with ongoing high vasopressor requirement.

### 7.3. Anticoagulants and the Glycocalyx

Anticoagulants have also been examined as treatments targeting the endothelium. Sepsis reduces the circulating levels of antithrombin-III (AT-III), which coincides with glycocalyx injury and derangement of the coagulation cascade leading to disseminated intravascular coagulation (DIC) [161]. Supplemental AT-III has been studied as a treatment for sepsis and DIC, though evidence is sparse [162]. A rat model of sepsis found that AT-III reduced leucocyte adhesion and rolling and plasma syndecan-1 concentrations. AT-III also maintained serum albumin concentrations and prevented hyperlactatemia, preserving microvascular function observed by SDF intravital microscopy [163]. Unfractionated heparin is a similar molecule to the glycocalyx component heparan sulfate, through which AT-III has its anticoagulant effect [164]. Heparin coating on CPB circuits to prevent endothelial damage and microvascular dysfunction has a protective effect [100]. The anticoagulant heparin has been used to antagonise cleavage of endothelial components and restore the integrity of the glycocalyx barrier [165].

## 8. Future Directions

The concept of personalised medicine arises from clinical enrichment, referring to patient subgroup selection of those who are more likely to respond to particular therapy as opposed to an unselected population, was mainly developed in the field of oncology. Its success in that field led to publication of Food and Drug Administration (FDA) guidelines and a statement of intent from the Obama Whitehouse to emphasize, prioritise and pursue enrichment strategies to develop novel therapies for diseases [166,167]. Prognostic enrichment is important in the design of clinical trials, identifying those patients more likely to encounter an outcome or complication, thus increasing the power of a study and reducing the required sample size [168]. Predictive enrichment requires exact knowledge of a biological mechanism to select patients that will respond to an intervention.

Prognostic enrichment has been used in ICU research to define acute respiratory distress syndrome phenotypes, which enhanced research uncovering therapeutic strategies. However, ICU syndromes such as sepsis lack a specific biological target, precluding predictive enrichment.

Microvascular imaging of glycocalyx behaviour and response to treatment could be the biological target needed to stratify patients into clinically relevant phenotypic groups. Using bedside diagnostics and imaging techniques together with machine learning and latent class analysis, better trials could be developed to identify effective therapies for patient subclasses.

Further tests on drugs like Sulodexide, a combination of heparin-sulfate like compound shown to regenerate the glycocalyx in a mouse model of sepsis, that also restores glycocalyx volume in diabetics, should be studied further in critical care [169]. FFP has shown benefit in animal models as well as models of haemorrhagic shock but there are no high quality studies of its effects restoring the glycocalyx in critical illness [170,171].

The glycocalyx spans all organs and therefore is exposed to variable rates of flow, as well having different thresholds for onset of glycocalyx damage leading to spatiotemporal uncoupling of insult and reaction. This could explain the differences in microcirculation measurements between organs seen in studies relating intestinal SDF microcirculation measurement to sublingual imaging [79]. Several studies noted the potential uncoupling of glycocalyx damage and the microcirculation variables. It is possible that the effect of glycocalyx damage undergoes a certain lag or that the recovery of the glycocalyx while bathed in septic plasma takes longer than in vitro. There could be immediate precipitation of glycocalyx change with delayed changes in perfusion, followed by prolonged repair of the glycocalyx. In studies of patients on CPB the microcirculation is affected almost immediately at the point of initiation with glycocalyx degradation markers not returning to baseline levels for 72 h [98,99]. We do not know at what point in sepsis the microcirculation becomes impaired, or similarly, how long it takes to improve. Further delineation of the relationship between the functional microcirculation and the detectable markers of glycocalyx damage could elucidate a novel therapeutic target in this syndrome.

## 9. Conclusions

Damage to the glycocalyx is present in a range of critical illness syndromes in the ICU and can be recognised in the lab and at the bedside. New technologies mean that real time monitoring of the glycocalyx could become important to patient treatment in the future. The development of new methods to examine the microcirculation at the bedside offers us the opportunity to understand the etiopathogenesis of ICU syndromes, advance patient monitoring and to personalise care in the ICU.

The development of new methods to examine the microcirculation at the bedside offers us the opportunity to advance patient monitoring and to personalise care in the ICU. Moving towards personalised medicine requires individualising treatments and enhancing our understanding of etiopathogenesis of ICU syndromes will improve patient outcomes. Phenotyping subsets of patients according to the reaction of their physiology to infection, inflammation, trauma or surgery will allow better clinical decision making. Large trials of therapeutic interventions have been affected by heterogeneity in ICU trials, combining the information from bedside imaging with biological tests will improve prognostication and therapy. Future research will benefit from combining systemic tests of endothelial integrity with bedside clinical evaluation of the microcirculation.
